# Impact of Tinted Lenses on Contrast Sensitivity, Color Vision, and Visual Reaction Time in Young Adults

**DOI:** 10.7759/cureus.48377

**Published:** 2023-11-06

**Authors:** Selva Seelan Samuel, Tamilselvan Pachiyappan, Samuel Livingstone Kumaran

**Affiliations:** 1 Optometry, Sri Ramachandra Institute of Higher Education and Research, Chennai, IND; 2 Public Health, Mangala College of Allied Health Sciences, Mangalore, IND

**Keywords:** color vision, young adults, tints, visual reaction time, contrast sensitivity

## Abstract

Background

Visual performance relies on essential functions such as contrast sensitivity, color vision, and visual reaction time. While studies have suggested that tinted lenses can enhance these visual functions, their effects on specific aspects remain insufficiently explored. Understanding the potential benefits and implications of tinted lenses is crucial for prescription considerations. This study aims to address this gap by evaluating the influence of tinted lenses on visual functions in young emmetropic adults.

Methodology

This prospective, cross-sectional study, approved by the institutional research and ethics committee, encompassed three key assessments, namely, contrast sensitivity using the Pelli-Robson chart, color vision with the D-15 test, and visual reaction time utilizing the Hit-the-dots method. The study involved 100 participants aged 18-30 years with normal vision, while individuals with color vision deficiencies, refractive errors, amblyopia, and strabismus were excluded. Assessments were conducted both with and without blue, yellow, brown, and gray-tinted lenses. Contrast sensitivity was quantified in logMAR units, color vision was evaluated using a color disc arrangement, and visual reaction time was measured with a 30-second reaction test employing an Asus Vivo Book. Data were analyzed using Friedman’s two-way analysis of variance by ranks, and specific differences were identified through the Dunn-Bonferroni method.

Results

The average age of the participants was 22.8 (2.8) years, with all participants exhibiting distance visual acuity of 6/6 and near visual acuity of N6. Contrast sensitivity values were consistent at 2.00 logMAR, indicating good contrast sensitivity, irrespective of the presence of tinted lenses. Notably, among the participants, yellow tint 50 (50%) emerged as the preferred choice for visualizing low-contrast optotypes. Friedman’s test revealed significant differences in color vision and visual reaction time between normal and tinted lenses (p < 0.001). The Dunn-Bonferroni test further indicated significant differences, particularly with yellow tint (p < 0.05) compared to other tints. In visual reaction time, significance (p < 0.05) was observed for all comparisons except between blue and gray, with the yellow tint demonstrating the best reaction time (median = 23) compared to other tints.

Conclusions

This research provides valuable insights into the impact of tinted lenses on contrast sensitivity, color vision, and visual reaction time in individuals with normal vision. Importantly, the use of yellow tint appears to enhance rapid reactions and contrast perception. Nevertheless, the selection of tint types should be thoughtfully tailored to individual occupational needs and personal preferences, taking into account their potential effects on color perception.

## Introduction

In the modern world, vision is a paramount element that facilitates the seamless execution of day-to-day activities unhindered by obstacles. Among the multifaceted dimensions of visual performance, contrast sensitivity (CS), color vision (CV), and visual reaction time (VRT) emerge as pivotal factors [1,2]. CS, the ability to discern minute and substantial pattern contrasts, profoundly influences visual recognition [3]. Notably, an individual with good visual acuity may still exhibit impaired CS, underscoring the significance of this facet. Similarly, CV involves the complex process of interpreting varying wavelengths that stimulate the retina to perceive diverse colors, a process unique to each individual [1,4]. VRT, on the other hand, is intricately linked to an individual’s alertness, reflecting the interplay between the central nervous system and the coordination between visual perception and motor response [2,5,6].

The classification of VRT encompasses three distinct categories, namely, simple reaction time (SRT), recognition reaction time (RRT), and cognitive reaction time (CRT). SRT denotes the temporal interval required for an individual to respond to a singular stimulus. In contrast, RRT encompasses a cognitive aspect where an individual selects the most appropriate response from multiple stimuli, the nature of which hinges on the stimuli’s characteristics. Finally, CRT encapsulates the process of recognizing stimulus significance, forging connections, and deploying knowledge to yield an optimal cognitive response congruent with the stimulus complexity [7].

Corrective devices, namely spectacles and contact lenses, hold widespread utility in optimizing visual functionality [1,8,9]. While clear lenses serve to rectify visual impairments, tinted lenses constitute a prominent subset of ophthalmic devices [1,10]. These lenses, available in an array of tints, such as gray, green, brown, violet, blue, and yellow, serve as filters that modify light intensity and spectral distribution [11]. Consequently, tints alleviate photosensitivity, enhance vision quality, and even find application in safeguarding eyes against solar radiation and augmenting visual perception [10,11]. Intriguingly, tinted lenses have exhibited the potential to enhance various aspects of visual performance, including CS, CV, and VRT, leading to their integration into diverse activities, such as sports, driving, and different environmental conditions [1,12].

In the realm of visual performance studies, sparse attention has been directed toward the influence of tints on CS, CV, and VRT. While existing research has noted enhancements in aspects such as CV with certain tints, contradictory findings have also emerged. For instance, the gray tint has been associated with improved CV, albeit without significant differentiation among various tint groups. In sports, tinted lenses have been linked to heightened CS and visual acuity, particularly in low-light conditions [12-15]. Notably, specific tints, such as yellow, have demonstrated favorable impacts on reaction time in select scenarios, such as low-contrast conditions and driving. However, this effect may vary with age, as observed in studies differentiating between young and elderly drivers [8,13,16].

Within professions that demand robust VRT, such as driving and sports, superior VRT is an anticipated attribute [17,18]. As such, comprehending the interplay of tints on CV, CS, and VRT assumes vital importance [7,19]. Furthermore, the multifaceted nature of these interactions, coupled with their potential repercussions on individual reaction times, warrants comprehensive exploration. Surprisingly, a dearth of literature exists on the subject of tints and their influence on visual functions, especially across diverse populations. Against this context, this investigation aimed to assess the effects of tinted lenses on visual functions within the young emmetropic adult population.

## Materials and methods

This research employed a prospective, cross-sectional study design. The study received approval from the Institutional Research and Ethics Committee under reference number CSP/21/MAY/94/323, and it adhered to the principles outlined in the Declaration of Helsinki. Within the study, three distinct measurement techniques were applied, namely, CS assessment using the Pelli-Robson chart, CV evaluation with the D-15 test, and VRT measurement using the Hit-the-dots method (specific assessment of RRT) designed by the University of Sports, Washington [20]. The study incorporated 50% light transmission spectacle tints in blue, yellow, brown, and gray. Vision Rx laboratory in India produced these ophthalmic spectacle lenses using CR-39 material. Additionally, the study included a subjective evaluation of participants’ tint preferences over the low-contrast optotypes of the CS chart.

Participation in the study was voluntary and required informed written consent from each participant. The study included a total of 100 participants, accounting for 200 eyes. Among these participants, there were 25 males and 75 females, all falling within the age range of 18 to 30 years. The sample size determination employed a t-test based on the Correlation: Point biserial model. A non-probability sampling technique was utilized for participant selection. The study included emmetropes with a visual acuity of 6/6 and trichromats, while individuals with CV deficiencies, ocular pathologies, systemic diseases, refractive errors, amblyopia, and strabismus were excluded.

**Figure 1:**
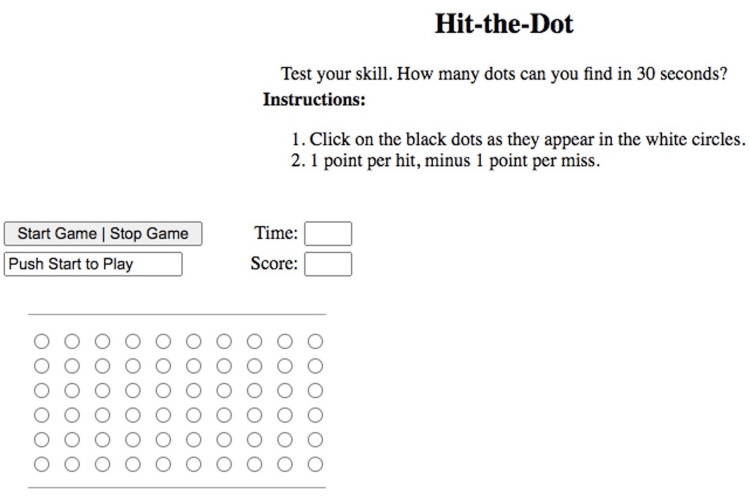
Screenshot of Hit-the-dots Reaction test. Measurement of recognition reaction time.

In an optometry clinical setting, comprehensive assessments were conducted both with and without (normal) the use of tinted lenses. Each participant underwent a meticulous ocular examination, followed by an evaluation of their visual acuity using a computer vision screen. CS measurements were recorded in logMAR units. The CV assessment consisted of a set of 15 colored discs, along with one reference disc, which participants were tasked with arranging in the correct order. Any errors made during this sequence arrangement were carefully documented. The Hit-the-dots Reaction test was administered using an Asus Vivo Book equipped with a CORE i5 processor. The display panel featured a 14-inch screen with a resolution of 1,920 x 1,080 pixels, set at a brightness level of 40%. The test was performed binocularly. During this test, subjects were required to locate and click on black dots that appeared within white circles (Figure 1). Each successful click earned participants one point, while any misses resulted in a deduction of points. The duration of the test was designed for 30 seconds. Before formal data collection commenced, participants underwent a preliminary trial run to acquaint themselves with the test procedure.

The collected data were analyzed with SPSS 23.0 version software (IBM Corp., Armonk, NY USA). The Kolmogorov-Smirnov test was used to check for normality. The statistical indicators included mean, standard deviation (SD), median, Interquartile range (IQR), frequency, and percentage for the variables. Friedman’s two-way analysis of variance by ranks was used to determine statistically significant differences in CV and VRT. The significance level was p < 0.05. Post-hoc test using the Dunn-Bonferroni method was conducted to identify specific differences. Kendall’s W was used to represent the agreement.

## Results

In this study, we enrolled 100 participants, totaling 200 eyes, consisting of 25 males and 75 females aged between 18 and 30 years. The average age and SD of the participants was 22.8 (2.8) years. All participants demonstrated normal unaided distance visual acuity of 6/6 and near visual acuity of N6.

The study found that CS values remained consistently at 2.00 logMAR, indicating good CS, both with and without tinted lenses. This suggests that the participants maintained good CS regardless of the presence of tinted lenses, with no observed reduction. When participants were asked about their preferences for tint colors in relation to the CS chart, 50 (50%) preferred yellow, 26 (26%) favored blue, 16 (16%) chose gray, and eight (8%) selected brown. Notably, yellow was the most preferred tint for visualizing low-contrast optotypes.

Friedman’s analysis of CV revealed a statistically significant difference between normal and tinted conditions (p < 0.001). The mean ranks were as follows: normal: 2.90, blue: 2.95, yellow: 3.21, brown: 2.98, and gray: 2.95. The Dunn-Bonferroni indicated a significant difference between yellow (p < 0.05) and the other tints, while the differences between the other tints were not significant (Table 1). Kendall’s W test indicated only slight agreement (0.059). The N (%) and total sum of errors observed with tinted lenses in CV assessment were as follows: blue: 4 (2%) with a total of nine errors, yellow: 24 (12%) with a total of 39 errors, brown: 6 (3%) with a total of 13 errors, and gray: 4 (2%) with a total of nine errors. The maximum error done by a participant was five with yellow, while with the other tinted lenses, it was three.

**Table 1:** Dunn-Bonferroni test for CV and VRT. *: P-value significant at <0.05. N: normal; B: blue; Y: yellow; Br: brown; Gr: gray; CV: color vision; VRT: visual reaction time

Multiple comparisons	P-value for CV	P-value for VRT
N-B	1.000	<0.001*
N-Y	<0.001*	<0.001*
N-Br	0.908	0.002*
N-G	1.000	<0.001*
B-N	1.000	<0.001*
B-Y	0.001*	<0.001*
B-Br	1.000	0.005*
B-G	1.000	1.000
Y-N	<0.001*	<0.001*
Y-B	0.001*	<0.001*
Y-Br	0.007*	<0.001*
Y-G	0.001*	<0.001*
Br-N	0.908	0.002*
Br-B	1.000	0.005*
Br-Y	0.007*	<0.001*
Br-G	1.000	0.028*
G-N	1.000	<0.001*
G-B	1.000	1.000
G-Y	<0.001*	<0.001*
G-Br	1.000	0.028*

The median VRT score for the normal was 21. The median scores improved with yellow, blue, and gray tints, indicating an increase in VRT with the use of these tints (Table 2). The Friedman’s test showed a statistically significant difference (p < 0.001) among normal and tinted conditions, with mean ranks as follows: normal: 1.66, blue: 3.10, yellow: 4.48, brown: 2.68, and gray: 3.08. The post hoc test revealed significance (p < 0.05) for all comparisons except between blue and gray (Table 1). Kendall’s W expressed a moderate agreement (0.448). It is noteworthy that all participants exclusively used their right hand during the VRT test, confirming the dominance of this hand.

**Table 2:** Visual reaction time scores among normal and tints. VRT: visual reaction time; SD: standard deviation; IQR: interquartile range

Parameters	Mean (SD)	Median (IQR)	Mode	Minimum score	Maximum score
VRT normal	20.98 (1.41)	21 (4)	21	19	23
VRT blue	21.96 (1.42)	22 (4)	20	20	24
VRT yellow	22.89 (1.45)	23 (5)	21	20	25
VRT brown	21.49 (1.11)	21 (3)	20	20	23
VRT gray	21.90 (1.41)	22 (4)	20	20	24

## Discussion

This research embarked on a comprehensive exploration of the impact of tinted lenses on CS, CV, and VRT in individuals with normal vision. To our knowledge, no prior study has undertaken such a thorough examination of these visual metrics within the scope of our analysis. Our findings consistently revealed intriguing patterns. Our observation begins with no discernible difference in CS when comparing with and without tinted lenses. All tints maintained good CS, aligning with their normal state. However, a notable disparity emerged in terms of CV, particularly with the yellow tint, which exhibited a significantly increased sum of errors compared to other tints. Furthermore, the VRT test showed significant improvements in reaction time with all tinted lenses chiefly with yellow and blue tints achieving the highest scores.

The significance of CS cannot be overstated, as it is essential for visual performance. Previous research demonstrated that 50% transmission tints exhibited good contrast function at 1.5 cycles per degree [1]. Our study corroborated these findings, confirming that the tints we tested did not negatively impact CS. Notably, our study did not explore variations in tint percentages, which have been associated with decreased CS in previous research [1]. While our study did not detect any worsening with tints, another study found that red and brown tints had the most negative influence on CS when compared with no tint, with green, gray, blue, and yellow following in descending order of impact [21]. Whereas a previous investigation into the average CS for various tint percentages within each group of refractive errors found no statistically significant differences in CS [22].

Any improvement or deterioration in CS could potentially be attributed to the tools and methods employed for CS measurement. While studies have utilized tools such as FACT (Functional Acuity Contrast Test) and PsychoWin 2.25 software for CS measurements, our study utilized the Pelli-Robson chart [1,11]. It is worth noting that factors such as room illumination and chart illumination could influence finer acuity measurements [23]. Advanced contrast tools, appropriate illumination, and varied tint transmission percentages might yield different results. Additionally, while our objective evaluation did not reveal CS differences, subjective preferences suggested that yellow tints were favored for low-contrast optotypes. This affinity for yellow tints may stem from their ability to filter blue light, enhancing contrast in low-light conditions, which could be relevant for indoor or outdoor activities, including sports. Consequently, properly measuring CS before prescribing tinted lenses with different percentages becomes crucial for various professions and pathologies to optimize visual performance [22].

Our study also shed light on the influence of tinted lenses on CV. Notably, yellow tints significantly altered CV compared to other tints. While our study used the D-15, other studies also utilized the American Optical Hardy Rand Rittler test and the Cambridge test as alternatives to CV testing [1]. Prior research has suggested that yellow and orange tints can reduce the ability to distinguish between colors, particularly affecting tritan-like CV abnormalities [11]. This may be the cause of our study’s finding that yellow 24(12%) tints considerably affect CV more than other tints. Yellow-tinted lenses have the potential to affect color perception, although the extent of these effects varies depending on the specific shade of yellow and other influencing factors. Yellow-tinted lenses are known to selectively filter out certain wavelengths of light, particularly those in the shorter blue region of the spectrum. Consequently, looking through yellow lenses can lead to a shift in the perception of some colors, making them appear less vibrant or altered [1,11,12,14]. In contrast, tints such as brown 6(3%), gray 4(2%), and blue 4(2%) had the least pronounced impact on color perception. The most noticeable alterations in our ability to perceive colors occur when using tints such as red and yellow, with subsequent changes observed in brown, green, gray, and blue hues. These findings align closely with our study results [21].

VRT, as a measure of response to visual stimuli, holds significance in various professions and sports that demand rapid responses [8,14]. While some studies have indicated that VRT tends to change with age, only a few have explored VRT with tinted lenses [24]. Given the observed heightened reaction times with the dominant hand in Hit-the-Dot tests, our study exclusively instructed participants to employ their dominant hand, ensuring consistency [7]. Surprisingly, the realm of VRT and its relationship with tinted lenses remains relatively unexplored in existing literature. A previous study, however, posited that yellow-tinted lenses enhance VRT [11]. Our investigation revealed intriguing results, with yellow-tinted lenses achieving the highest score of 25. In comparison, other tints, such as blue, brown, and gray, showed improvements over the normal. This finding resonates with previous research which delved into the impact of the color yellow on driving responses. Their results revealed that young drivers exhibited improved response times when exposed to yellow, whereas elderly drivers did not demonstrate the same level of enhancement [11,13].

Multiple factors have been proposed to influence VRT, including visual acuity, CS, CV, illumination, attention, age, and fatigue [25,26]. The pivotal role of VRT in driving safety has garnered significant attention in recent studies. Yellow-tinted lenses, in particular, have emerged as a distinctive lens variant of interest. These lenses efficiently filter out blue light rays and possess high luminous transmittance, especially at approximately 550 nm, a wavelength highly sensitive to the human eye. This unique optical property may explain the observed increase in reaction time when wearing yellow-tinted lenses [14]. Furthermore, certain studies have noted that yellow filters can effectively mitigate chromatic aberration. Additionally, it has been documented that the presence of a yellow filter induces greater pupil dilation compared to a matched neutral-density filter. This variance in pupil response could partially account for the perceived enhancement in brightness reported by individuals using yellow filters [27]. Consequently, future studies in this domain must consider these multifaceted factors to provide a more comprehensive understanding of the effects of tinted lenses on VRT.

Our study has certain strengths; comprehensive investigation of CS, CV, and VRT with tinted lenses, novelty in terms of comparison of the variety of tints with these visual metrics, clinical setting with standardized illumination of 500 lux, inclusion of both objective and subjective measures considering both clinical outcomes and participant experiences, and importance in comprehending how tints affect VRT specifically RRT. Some of our study’s limitations are limited sample size, lack of a diverse population (wider age group and refractive error), utilization of different tint percentages, and lack of long-term assessment of how visual performance may adapt or change over time with consistent tint usage. Although our study provides significant insights into the multidimensional impacts of tinted lenses on several aspects of visual function, particularly in an emmetropic and young population, future research should explore these visual metrics (CS, VRT, and CV) by considering the limitations.

## Conclusions

This research provides valuable insights into the effects of tinted lenses on CS, CV, and VRT in individuals with normal vision. In summary, our study shows that tints can impact various visual functions, especially yellow ones, which may be beneficial for quick RRT and good contrast and their impact on color perception should be considered. These findings hold particular relevance for individuals engaged in occupations and sports where rapid reactions and precise CV are paramount. However, the choice of tints should be carefully prescribed based on specific occupational needs and preferences.

## References

[1] Shaik M, Majola P, Nkgare L, Nene N, Singh C, Hansraj R, Rampersad N (2013). The effect of tinted spectacle lenses on contrast sensitivity and colour vision. S Afr Optom.

[2] Kalyanshetti SB (2014). Effect of colour of object on simple visual reaction time in normal subjects. J Krishna Inst Med Sci.

[3] Lee JE, Stein JJ, Prevor MB, Seiple WH, Holopigian K, Greenstein VC, Stenson SM (2002). Effect of variable tinted spectacle lenses on visual performance in control subjects. CLAO J.

[4] Noticewala V, Shastri M (2017). A study of contrast sensitivity changes in normal individual and diabetic patients with and without diabetic retinopathy. Int J Res Med Sci.

[5] Mohanraj S, Karthikeyan A (2017). Comparison of visual reaction time in myopic subjects with emmetropic subjects. Natl J Physiol Pharm Pharmacol.

[6] Amini Vishteh R, Mirzajani A, Jafarzadehpour E, Darvishpour S (2019). Evaluation of simple visual reaction time of different colored light stimuli in visually normal  students. Clin Optom (Auckl).

[7] Badau D, Baydil B, Badau A (2018). Differences among three measures of reaction time based on hand laterality in individual sports. Sports (Basel).

[8] Patel Z, Mazibuko S, Rampersad N, Moodley S, Paruk H, Nsele N, Hansraj R (2019). The effect of tints on distance stereoacuity under varying retinal illuminations. Afr Vision Eye Health.

[9] Harries P, Hall R, Ray N, Stein J (2015). Using coloured filters to reduce the symptoms of visual stress in children with reading delay. Scand J Occup Ther.

[10] Simmers AJ, Gray LS, Wilkins AJ (2001). The influence of tinted lenses upon ocular accommodation. Vision Res.

[11] de Fez MD, Luque MJ, Viqueira V (2002). Enhancement of contrast sensitivity and losses of chromatic discrimination with tinted lenses. Optom Vis Sci.

[12] Aoki K, Kohmura Y, Murakami S, Someya Y (2015). Effects of yellow-tinted lenses on visual attributes related to sports activities and daily life in late middle-aged adults. Central Eur J Sport Sci Med.

[13] Doroudgar S, Chuang HM, Perry PJ, Thomas K, Bohnert K, Canedo J (2017). Driving performance comparing older versus younger drivers. Traffic Inj Prev.

[14] Kohmura Y, Murakami S, Aoki K (2013). Effect of yellow-tinted lenses on visual attributes related to sports activities. J Hum Kinet.

[15] Lingelbach B, Jendrusch G (2005). Contrast enhancing filters in ski sports. J ASTM Int.

[16] Hall R, Ray N, Harries P, Stein J (2013). A comparison of two-coloured filter systems for treating visual reading difficulties. Disabil Rehabil.

[17] Mehta M, Ramkissoon P, Bhagwanjee AM (2007). A comparison of the effect of reduced illumination and tinted lenses on stereospsis at near. Afr Vision Eye Health.

[18] Balakrishnan G, Uppinakudru G, Girwar Singh G, Bangera S, Dutt Raghavendra A, Thangavel D (2014). A comparative study on visual choice reaction time for different colors in females. Neurol Res Int.

[19] Nangia V, Jonas JB, Sinha A, Gupta R, Agarwal S (2011). Visual acuity and associated factors. The Central India Eye and Medical Study. PLoS One.

[20] (2023). Chudler EH. Hit the Dot. Recognition Reaction Time Test. University of Washington. https://faculty.washington.edu/chudler/java/dottime.html.

[21] Singh S, Kajol Kajol, Singh G (2023). The effect of different tints on colour vision and contrast sensitivity. Int J Innov Sci Res Technol.

[22] Kalikivayi V, Kalikivayi L (2021). Influence of various densities of yellow and pink tinted spectacles on contrast sensitivity. J Exp Clin Ophthalmol.

[23] Tidbury LP, Czanner G, Newsham D (2016). Fiat Lux: the effect of illuminance on acuity testing. Graefes Arch Clin Exp Ophthalmol.

[24] Anstey KJ, Wood J, Lord S, Walker JG (2005). Cognitive, sensory and physical factors enabling driving safety in older adults. Clin Psychol Rev.

[25] Simunovic MP (2010). Colour vision deficiency. Eye (Lond).

[26] Owsley C, Ball K, McGwin G Jr, Sloane ME, Roenker DL, White MF, Overley ET (1998). Visual processing impairment and risk of motor vehicle crash among older adults. JAMA.

[27] Lacherez P, Saeri AK, Wood JM, Atchison DA, Horswill MS (2013). A yellow filter improves response times to low-contrast targets and traffic hazards. Optom Vis Sci.

